# Recurrence after hepatic resection in colorectal cancer liver metastasis -Review article-

**Published:** 2015

**Authors:** B Lintoiu-Ursut, A Tulin, S Constantinoiu

**Affiliations:** * ”Prof. Dr. Agrippa Ionescu” Clinical Emergency Hospital, Bucharest, Romania; ** Department of Anatomy, “Carol Davila” University of Medicine and Pharmacy, Bucharest, Romania; *** Department of General and Esophageal Surgery, ”Carol Davila” University of Medicine and Pharmacy; “Sf. Maria” Clinical Hospital, Bucharest, Romania

**Keywords:** colorectal cancer, hepatic metastasis, liver resection

## Abstract

The outcomes and management of colorectal liver metastasis have undergone many changes. The incidence of recurrence after liver resection for hepatic metastasis remains very high. Liver resection, which provides the only curative treatment, is believed to have improved the long-term outcome of these patients. However, the management and outcomes of patients with colorectal liver metastasis have greatly improved in the last decade, suggesting that the current use of aggressive multimodality treatments, including surgical resection combined with modern chemotherapeutic regimens, effectively prolong the life expectancy of these patients.

## Introduction

Colorectal cancer is a major health problem. It is the third most frequent cancer in men (764 000 cases/ year; 10% of the total incidence) and the second in women (614 000 cases/year; 9,2% of the total incidence). It is estimated that, in 2012, 1 360 000 people were diagnosed with colorectal cancer and 694 000 died from this disease worldwide. However, the incidence of colorectal cancer in 100 000 individuals decreased from 60,5 in 1976 to 46,4 in 2005. Moreover, the mortality decreased with almost 30% from 1990 until 2007, probably due to screening programs and thus earlier diagnosis and also due to better therapeutic approaches [**1**-**4**]. In USA, an ongoing program has the final goal of achieving 80% colorectal screening rates by 2018 [**5**]. 

## Liver metastasis

More than 50% of the patients with colorectal cancer (CRC) have or will develop metastasis, with a quarter having distant metastatic disease at the time of diagnosis, most frequently in the liver [**6**]. Liver metastasis is the leading cause of cancer-related morbidity and mortality in colorectal cancer. The only potentially curative treatment for liver metastasis is liver resection, but only 15% to 20% of the patients are suitable for surgical resection. Long-term survival is expected with the complete curative resection, even in patients with initially unresectable metastasis [**6**].

In the last decade, the incidence of hepatic surgery for colorectal metastasis has increased, with a 5-year survival up to 60% in recently published studies [**7**,**8**]. Primary liver resection can achieve long-term survival in 25% to 30% of the patients with hepatic colorectal metastasis as compared to 2%, the 5-year survival with the natural history of the disease [**7**,**9**]. The 5-year overall survival rate following resection has been higher in the last years due to the improvement in patient selection, perioperative care and better surgical techniques [**10**].

Methods to increase the proportion of patients who are candidates for complete surgical treatment include preoperative portal vein embolization/ ligation, two-stage resection, neoadjuvant chemotherapy, association of radiofrequency ablation to surgery [**10**,**11**]. Portal vein embolization or ligation may provide the adequate future remnant liver volume. They induce hypertrophy in the future remnant liver in order to prevent liver failure after major hepatectomy [**6**]. In selected patients, the metastasis can be down-staged by systemic chemotherapy with or without molecular targeted therapy, so that liver resection can be completed. The development of efficient molecular-targeted drugs, such as cetuximab or bevacizumab, has opened new perspectives in the treatment of resectable and unresectable liver metastasis. In patients with KRAS wild-type tumors, chemotherapy with cetuximab was demonstrated to achieve high response rates [**12**,**13**].

## Recurrence of the disease

After liver resection, postoperative adjuvant chemotherapy is recommended for all patients, unless the patient’s physical status is unsuitable for chemotherapy or patients are unwilling to receive this therapy [**8**].

Nevertheless, tumor recurrence after curative resection of colorectal metastasis remains a major problem. 50% to 75% of the patients develop a recurrence of the disease within two years [**8**,**14**]. Surgical resection of the metastatic lesions with curative intent is also the treatment of choice for recurrence after liver resection for colorectal cancer liver metastasis. 

The strategy for the treatment of recurrent colorectal cancer after liver resection is the same as that for the initial colorectal cancer metastasis and depends on the consensus of a multidisciplinary committee [**8**,**11**,**15**]. 40% of such patients are candidates for further surgery. There are also patients reported to have undergone third hepatic procedures for colorectal liver metastasis (CRLM) [**7**].

Several factors were associated with recurrence after hepatectomy in colorectal metastasis: primary colorectal tumor stage, time interval to the appearance of metastasis, preoperative CEA level, adjuvant/ neoadjuvant chemotherapy. Recently, several studies identified pathologic prognostic markers of hepatic tumors predicting the prognosis of these patients. The dedifferentiation and tumor infiltrating inflammation of metastatic lesions are considered predictive factors of tumor recurrence in colorectal liver metastasis [**14**]. The mechanism of recurrence may include inadequate margin or missed lesions from the first operation or natural progression of micrometastatic disease from the primary tumor. 

The time interval from the initial colorectal surgery to first hepatic surgery has a median 11 months and between first and second hepatic procedures a median of 12 months. The morbidity rates are similar to those seen in primary hepatic resections. The most common postoperative complications reported are: hemorrhage, infections, biliary leak, transient hepatic failure. The repeat hepatic resection is a more difficult operation due to adhesions and intrahepatic anatomic variations induced by prior resections. Also, hepatic parenchymal hypertrophy and friability increases the difficulty of the surgery [**7**].

## Repeated hepatectomy

Repeated hepatectomy has been found a feasible treatment for recurrent CRLM. The 5-year overall survival rate was estimated to be of up to 55%. Many studies reported that repeat hepatectomy for recurrent CRLM is a safe procedure and improves the survival outcomes [**16**-**18**]. Number, size, location of lesions, metachronous CRLM, high CEA levels and extrahepatic metastasis have been reported to be risk factors of poor prognosis after repeat hepatectomy for recurrent CRLM. Radiofrequency ablation may be added to hepatectomy in order to preserve liver parenchyma. Liver transplantation is another option for unresectable CRLM and has comparable oncological outcomes as liver resection for resectable CRLM [**16**].

Predictors for CRC recurrence following liver resection remain an issue of great concern. The presence of multiple hepatic metastatic tumors, and specifically more than four, seems to be a very important risk factor for CRC recurrence after liver resection. This condition should not be considered a contraindication for liver resection and the safety of hepatic resection and more effective adjuvant chemotherapy support the concept of an aggressive surgical approach for these patients [**8**,**15**].

It is recommended that the follow-up for CRC recurrence include the measuring serum carcinoembryonic antigen (CEA) levels, liver ultrasonography one month after liver resection and every three months thereafter. Computed tomography or magnetic resonance imaging should be performed at yearly intervals or whenever CRC recurrence is suspected [**8**].

The conditions for the second resection are the same as those used to select patients for the first resection: no comorbidities to preclude surgery, all known disease is resectable, adequate margins (R0) are obtainable, preservation of a sufficient volume of liver [**7**,**8**]. Results of repeat resection are similar to those of the initial hepatectomy. The second hepatic resection can be performed safely with the same risks and the same survival rates as the first resection. So, repeat hepatic surgery is the best treatment option for selected patients with recurrent colorectal hepatic metastasis and the only chance for cure [**7**].

## Conclusions

Along with the advances in perioperative care, resecability and the overall survival of the patients with colorectal liver metastasis have shown remarkable improvements. However, the recurrence of hepatic metastasis after liver resection remains a concern worldwide. The treatment strategies regarding liver metastasis in colorectal cancer have changed along with the advancements in systemic therapy and surgical techniques in the last decade, thus improving the overall survival. There is no consensus regarding which therapeutic protocol is the best for the prevention of colorectal cancer recurrence after liver resection. Despite distant metastasis, colorectal cancer is one of the few malignancies for which patients with metastasis confined to a single organ may obtain long-term survival through a multidisciplinary approach. Due to the beneficial results of surgical resection for recurrent lesions, it is essential to regularly follow-up patients in the first years after liver resection. 

**Acknowledgements**

This paper is supported by the Sectoral Operational Programme Human Resources Development (SOP HRD), financed from the European Social Fund and by the Romanian Government under the contract number POSDRU/159/1.5/S/137390”.
